# Revealing stromal and lymphoid sources of *Col3a1*-expression during inflammation using a novel reporter mouse

**DOI:** 10.1093/discim/kyac008

**Published:** 2022-11-21

**Authors:** Larissa C da Rosa, Hannah E Scales, Sangeet Makhija, Katie Sutherland, Robert A Benson, James M Brewer, Paul Garside

**Affiliations:** School of Infection and Immunity, College of Medical, Veterinary, and Life Sciences, University of Glasgow, Glasgow G12 8TA, UK; School of Infection and Immunity, College of Medical, Veterinary, and Life Sciences, University of Glasgow, Glasgow G12 8TA, UK; School of Infection and Immunity, College of Medical, Veterinary, and Life Sciences, University of Glasgow, Glasgow G12 8TA, UK; School of Infection and Immunity, College of Medical, Veterinary, and Life Sciences, University of Glasgow, Glasgow G12 8TA, UK; School of Infection and Immunity, College of Medical, Veterinary, and Life Sciences, University of Glasgow, Glasgow G12 8TA, UK; School of Infection and Immunity, College of Medical, Veterinary, and Life Sciences, University of Glasgow, Glasgow G12 8TA, UK; School of Infection and Immunity, College of Medical, Veterinary, and Life Sciences, University of Glasgow, Glasgow G12 8TA, UK

**Keywords:** collagen type III, *Col3a1*, fibrosis, footpad inflammation, reporter mouse

## Abstract

One of the earliest signs of dysregulation of the homeostatic process of fibrosis, associated with pathology in chronic conditions such as rheumatoid arthritis, is the overexpression of collagen type III (COL-3). Critically, there is still relatively little known regarding the identity of the cell types expressing the gene encoding COL-3 (*Col3a1*). Identifying and characterizing *Col3a1*-expressing cells during the development of fibrosis could reveal new targets for the diagnosis and treatment of fibrosis-related pathologies. As such, a reporter mouse expressing concomitantly *Col3a1* and mKate-2, a fluorescent protein, was generated. Using models of footpad inflammation, we demonstrated its effectiveness as a tool to measure the expression of COL-3 during the repair process and provided an initial characterization of some of the stromal and immune cells responsible for *Col3a1* expression.

## Graphical Abstract

**Graphical Abstract:**
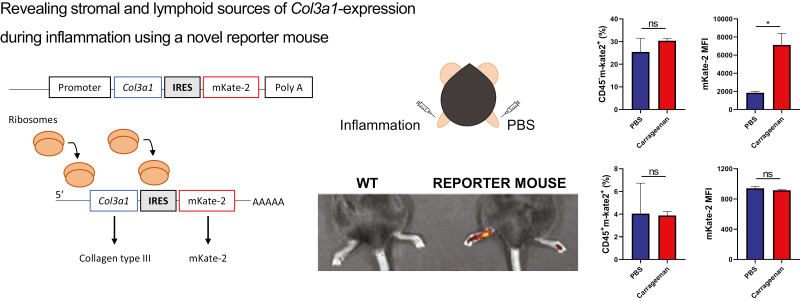

## Introduction

Overexpression and accumulation of extracellular matrix (ECM) components, like collagen type III (COL-3), is associated with the fibrosis that causes disruption of tissue architecture and function [1, 2] and can affect a wide variety of tissues. As a key mechanism in the progression of diseases with high mortality rates, such as cancer, pulmonary fibrosis, and heart failure, it is estimated to contribute to 45% of all deaths in the developed world [3, 4]. Moreover, it has an impact on mobility, as fibrosis is also involved in the joint deformation, weakening, and pain reported in osteoarthritis, tendinopathy, and rheumatoid arthritis (RA) [5, 6].

Fibrosis is often detected only at advanced stages, as biomarkers for the aberrant expression of collagen are yet to be discovered and tested [7]. Consequently, it is rarely targeted therapeutically. Only two antifibrotic drugs (Nintedanib and Pirfenidone) have been approved for the treatment of idiopathic pulmonary fibrosis and Sclerosis-associated Interstitial Lung Disease (SSc-ILD), and they are currently being tested in other fibrotic diseases [8, 9]. Studies demonstrated that nintedanib, a tyrosine kinase inhibitor, targets receptors for molecules such as fibroblast growth factor (FGF), platelet-derived growth factor (PDGF), and transforming growth factor-beta (TGF-β) [10].

One of the earliest signs of fibrosis is increased deposition of COL-3, reported in different organs, such as lung, renal, liver, and cardiac fibrosis [11] and arthritic diseases like tendinopathy [12]. COL-3 is encoded by gene *Col3a1* in mice [11], although little is known about the cells expressing more *Col3a1* and driving the levels of COL-3 protein up. This knowledge will be important for the design of new biomarkers and therapeutics to identify and treat fibrotic processes earlier.

To examine *Col3a1*-expressing cells and their potential as a biomarker/therapeutic target, we developed a reporter mouse simultaneously expressing the *Col3a1* gene and a fluorescent protein, mKate-2, under the control of the *Col3a1* promoter. Our aim in this study was to validate the use of the reporter mouse by investigating *Col3a1* expression throughout the development of two different models of inflammation.

## Materials and methods

### Mice

The reporter mouse, developed in collaboration with GenOway, was created by insertion of an Internal Ribosome Entry Site (IRES)—mKate2 report downstream of the *Col3a1* STOP codon in a C57BL/6 mouse background. Mice were bred and housed in specific pathogen free (SPF) facilities at the University of Glasgow’s Central Research Facility. Wildtype (WT) C57BL/6 mice were purchased from Envigo (Bicester, UK). All mice were maintained under standard animal house conditions, and all procedures were conducted in accordance with UK Home Office regulations.

### Acute footpad inflammation

Footpad inflammation was induced by injecting 50 µL of 1% carrageenan (Sigma–Aldrich) into one footpad, and the contralateral with phosphate-buffered saline (PBS) as described previously [13].

### OVA-induced delayed type hypersensitivity (DTH) model

DTH was induced and assessed as described previously [14].

### *In vivo* imaging system (IVIS)

Mice were anesthetized prior to imaging with IVIS Spectrum (PerkinElmer; MA, USA). Excitation and emission bandpass filters were 570/30 nm and 640/20 nm, respectively. Exposure time was 1 second, with medium binning and a f-stop of 1. Images were analysed using Living Image (PerkinElmer; MA, USA). A region of interest (ROI) was drawn manually around each footpad and the average of radiance efficiency {[p/s/cm²/sr]/[µW/cm²]} calculated.

### Tissue processing

Mice were euthanized, and footpads were collected. Single-cell suspensions were prepared by teasing tissues apart and digesting with 2.5 mg/mL collagenase D (Roche/Sigma–Aldrich) for 20 minutes at 37°C. Digestion was stopped with complete medium {RPMI + 10% foetal bovine serum [FBS]; Gibco}, samples were homogenized in a tissue dissociator (Miltenyi) and passed through a 70 µm cell strainer (BDBiosciences).

### Flow cytometry

Single-cell suspensions were resuspended in FACS buffer (PBS, 2% FBS, and 2 mM EDTA). For distinction of live cells, fixable viability dye (eFluor506, eBioscience) was added and incubated for 15 minutes, at 4°C. After centrifuging, non-specific FcR binding was blocked for 10 minutes at 4°C and then cells were incubated with fluorochrome-conjugated antibodies for 30 minutes, at 4°C. The markers used were: CD45 (BUV395; BD Bioscience), CD11b (FITC or AF700; BD Bioscience), Lymphocyte antigen six complex locus G6D (Ly6G. APC or BV421; BD Bioscience and Biolegend, respectively), Podoplanin (FITC; Biolegend), and CD140a (APC; Biolegend). Data were acquired on BD LSRFortessa (BD Biosciences) and analysed using FlowJo 10 software (Tree Star).

### Data analysis

Average radiance efficiency, footpad measurements, and flow cytometry results were represented as the mean ± standard deviation (SD). Data were compared by one-way- or two-way-ANOVA, followed by post hoc Tukey’s test, for multiple comparisons, and unpaired *t*-test with Welch’s correction for comparisons between two groups. The *P*-value adopted was *P* < 0.05, and statistical analysis was performed using Prism 8.3 (GraphPad).

## Results

### The mKate-2 fluorescence signal can be detected and is higher in homozygous mice

**Figure 1:**
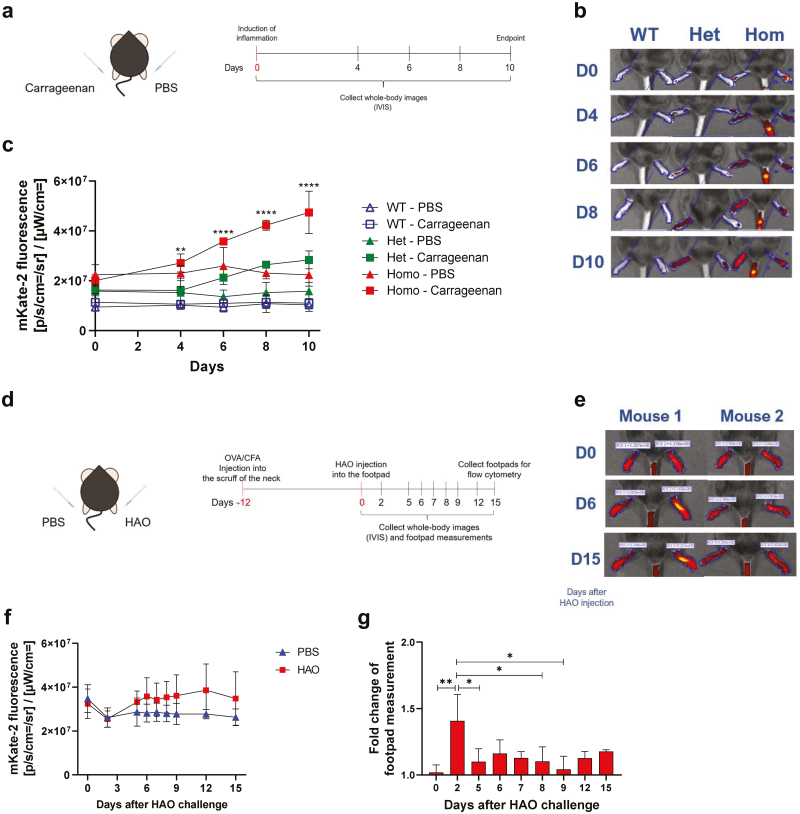
mKate-2 expression in two models of inflammation. (**a**) to examine differences in mKate-2 expression between heterozygous and homozygous *Col3a1*mKate-2 mice and wild-type mice were injected with 1% carrageenan in the left and PBS in the right footpad. mKate-2 fluorescence was measured on IVIS on days 0, 4, 6, 8, and 10. (**b**) Representative IVIS images of mouse footpads. Fluorescence was excited, and emission was detected on 570 nm and 640 nm wavelength filters, respectively. (**c**) Quantification of mKate-2 fluorescence, as the average of radiant efficiency. (**d**) For the DTH model, *Col3a1*mKate-2 mice were first injected with ovalbumin (OVA) emulsified in CFA subcutaneously into the scruff. Twelve days later, footpads were injected with either PBS (left footpads) or HAO (right footpads). Mice were, then, monitored for 15 days. (**e**) Representative IVIS images of mouse footpads over 15 days. (**f**) Quantification of mKate-2 fluorescence, detected by IVIS, as the average of radiant efficiency. (**g**) Fold change of the footpad thickness in footpads (measured daily with a calliper) injected with HAO, in relation to footpads injected with PBS. Data are represented as the mean ± SD. Statistical significance was determined using one-way or two-way ANOVA, followed by Tukey’s multiple comparisons test. *P < 0.05; **P < 0.01; ****P < 0.0001 and *n* = 3 per group, done over one experiment.

To establish whether *Col3a1* expression was detectable as mKate-2 fluorescence and whether there were differences between heterozygous and homozygous reporter mice, an acute transient inflammation was chosen. Inflammation caused by 1% carrageenan peaks at 24 hours [13]. We followed inflammation progression and resolution for 10 days (Fig. 1a). IVIS imaging revealed that mKate-2 fluorescence increased with time and was significantly higher in both homozygous and heterozygous reporter mice injected with carrageenan, compared to PBS, with the highest values observed in homozygous mice. No increased fluorescence was observed in the WT mice (Fig. 1b and c). All subsequent experiments were performed using homozygous *Col3a1*mKate-2 mice.

### Increased mKate-2 fluorescence due to inflammation is localized at the site of injection

To determine the utility of the reporter for assessing different types of inflammation, two models of footpad inflammation were employed: (i) we repeated the carrageenan-induced inflammation confirming the findings from Fig. 1c (data not shown), and (ii) an OVA-induced DTH, mediated by adaptive immunity. For the latter, *Col3a1*mKate-2 reporter mice were immunized with OVA/CFA and 12 days later challenged in the footpads with either heat-aggregated OVA (HAO) or PBS (Fig. 1d). Subsequently, footpad fluorescence and thickness were assessed. As previously, increased fluorescence occurred locally in footpads (Fig. 1e) and was higher following HAO injection (Fig. 1f). Interestingly, in footpads where the DTH process was occurring, fluorescence intensity was inversely proportional to footpad thickness (Fig. 1g). Thus, mKate-2 fluorescence (i.e. *Col3a1* expression) increased as the DTH was resolving.

### mKate-2 is expressed by both stromal and immune cells

**Figure 2:**
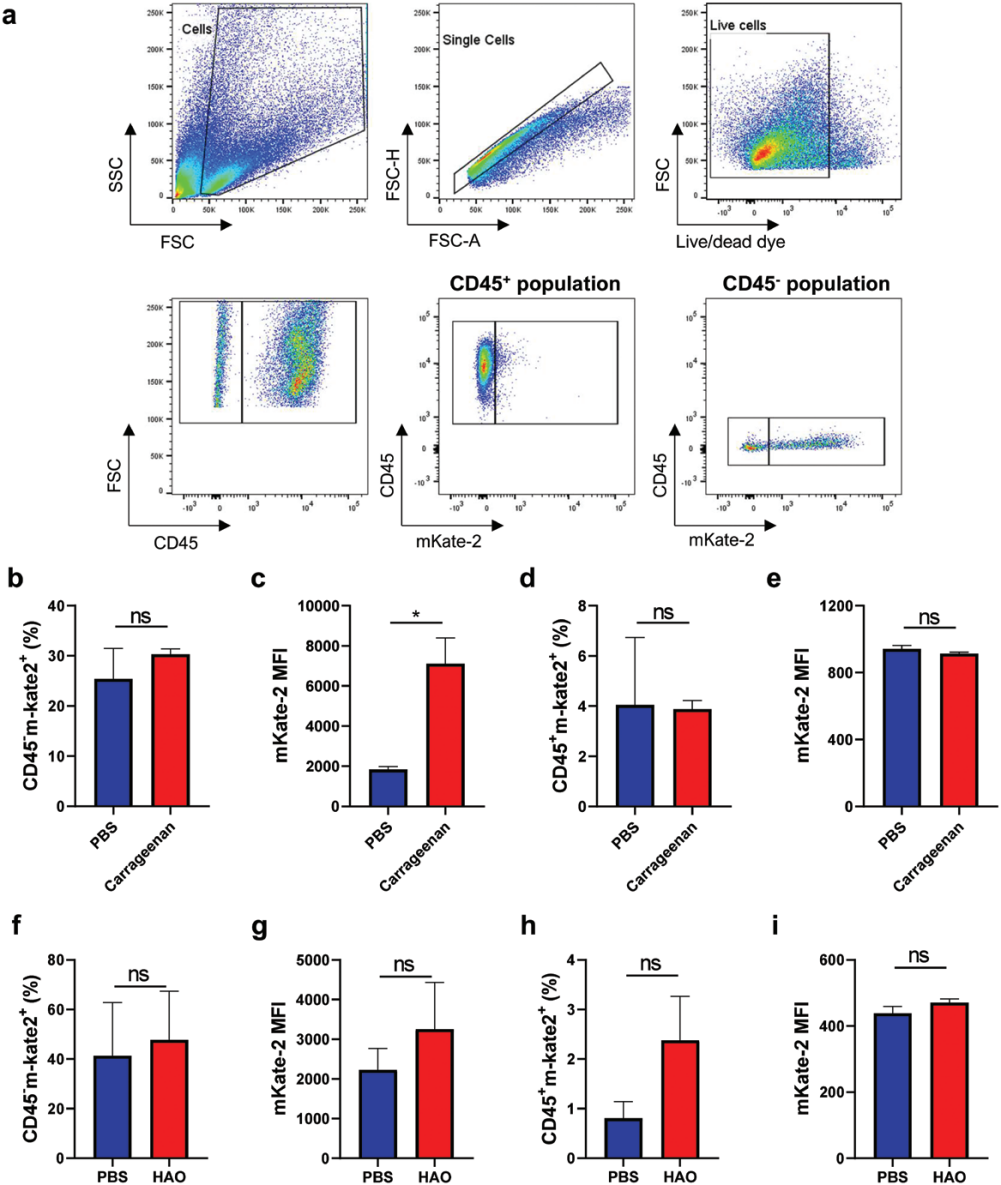
Flow cytometry characterization of *Col3a1*-expressing cells. Eight or 15 days after the carrageenan or HAO injection, respectively, footpads were collected, processed to single cell suspensions, and analysed by flow cytometry. (**a**) representative flow cytometry gating for mKate-2^+^ cells. Single, live cells were distinguished as immune or stromal cells by the presence or absence, respectively, of CD45 and examined for the expression of mKate-2. For the carrageenan experiment—(**b**) percentage of stromal cells (CD45^−^) expressing mKate-2. (**c**) Median of mKate-2^+^ fluorescence intensity in CD45^−^ cells. (**d**) Percentage of immune cells (CD45^+^) expressing mKate-2. (**e**) Median of mKate-2^+^ fluorescence intensity in CD45^+^ cells. For the DTH experiment—(**f**) percentage of stromal cells (CD45^−^) expressing mKate-2. (**g**) Median of mKate-2 fluorescence intensity in CD45^−^ cells. (**h**) Percentage of immune cells (CD45^+^) expressing mKate-2. (**i)** Median of mKate-2 fluorescence intensity in CD45^+^ cells. Negative control is represented in blue and inflammation-induced footpads in red. Data is represented in bar graphs as the mean ± SD of all mice in the group. Statistical significance was determined using *t*-test with Welch’s correction. *P < 0.05; ns = non-significant and *n* = 3 per group, done over one experiment.

As COL-3 and mKate2 are expressed as separate proteins, another advantage of the reporter mouse is that it allows us to identify cells expressing *Col3a1*. Therefore, footpads were collected, and cells were phenotyped by flow cytometry. Immune and stromal cells were distinguished by CD45 presence or absence and examined for mKate-2 expression (Fig. 2a). A representative of mKate-2 expression on a WT mouse is shown in Supplementary Fig. S1. In carrageenan- or PBS-injected footpads, immune and stromal cells expressed *Col3a1*, but there was a difference in the relative fluorescence contribution of these populations. While the proportion of CD45^−^ cells expressing mKate-2 was not significantly different (Fig. 2b), the mKate-2 fluorescence intensity (MFI) was higher in carrageenan-injected footpads (Fig. 2c), indicating increased expression of mKate-2 by these cells. In contrast, neither the frequency nor the MFI of CD45^+^mKate-2^+^ cells in carrageenan-injected footpads were altered (Fig. 2d and e). Considering the dynamics of mKate-2 expression seen on Fig. 1f, for future experiments DTH final timepoint should be day 12, when the fluorescence intensity peaked. The difference between PBS- and HAO-injected footpads could, then, be more expressive.

In the DTH model, the percentages of stromal cells expressing mKate-2 (Fig. 2f) and the MFI of mKate-2 (Fig. 2g) were similar between the groups. A higher frequency, although not significant (ns), of CD45^+^mKate-2^+^ cells was observed in mice injected with HAO (Fig. 2h), while the expression was similar (Fig. 2i), in comparison to footpads injected with PBS.

### The majority of cells expressing *Col3a1* in the footpads are podoplanin-expressing fibroblasts

Fibroblasts are considered the main collagen-producing cells, including COL-3 [2]. They are heterogeneous, and many markers are used to identify them. We chose two commonly used markers, CD140a and Podoplanin, to identify fibroblasts in the mKate2^+^ stromal population.

**Figure 3:**
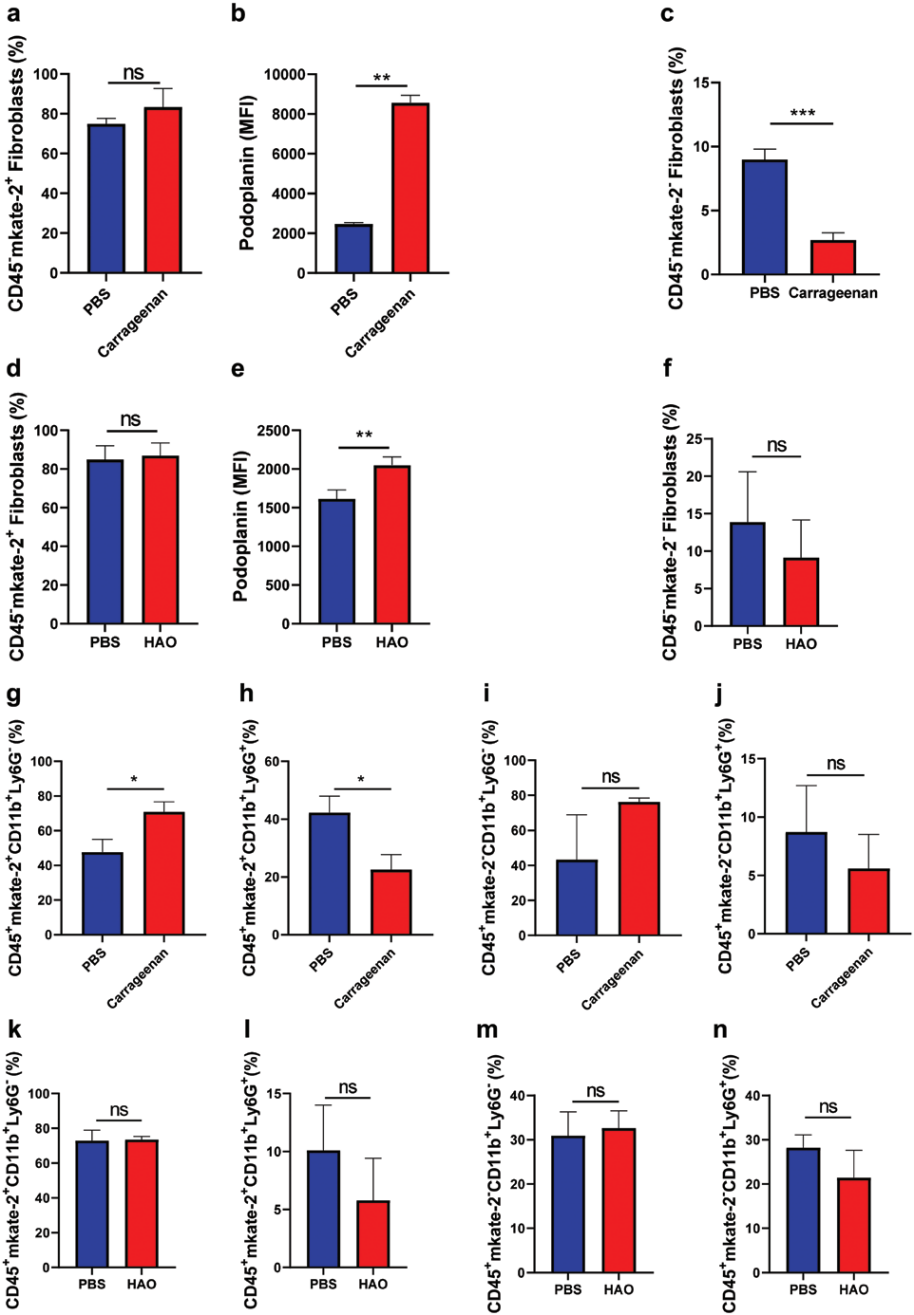
Flow cytometry characterization of stromal and immune cells expressing Col3a1. Eight or 15 days after the carrageenan or HAO injection, respectively, footpads were collected, processed to single cell suspensions, and analysed by flow cytometry. Stromal cells in carrageenan-induced inflammation—(**a**) percentage of CD45^−^mKate-2^+^ cells expressing PDPN and CD140a^+^ concomitantly. (**b**) PDPN^+^ median fluorescence intensity in CD45^−^mKate-2^+^PDPN^+^CD140a^+^ cells. (**c**) Percentage of CD45^−^mKate-2^−^ cells expressing PDPN and CD140a^+^ concomitantly. Stromal cells in the DTH model—(**d**) percentage of CD45^−^mKate-2^+^ cells expressing PDPN and CD140a^+^ concomitantly. (**e**) PDPN^+^ median fluorescence intensity in CD45^-^mKate-2^+^ PDPN^+^CD140a^+^ cells. (**f**) Percentage of CD45^−^mKate-2^−^ cells expressing PDPN and CD140a^+^ concomitantly. Immune cells in carrageenan-induced inflammation—(**g**) percentage of CD45^+^mKate-2^+^ cells expressing CD11b^+^Ly6G^−^ (monocytes/macrophages). (**h**) Percentage of CD45^+^mKate-2^+^ cells expressing CD11b^+^Ly6G^+^ (neutrophils). (**i**) Percentage of CD45^+^mKate-2^−^ cells expressing CD11b^+^Ly6G^−^. (**j**) Percentage of CD45^+^mKate-2^−^ cells expressing CD11b^+^Ly6G^+^. Immune cells in the DTH experiment—(**k**) percentage of CD45^+^mKate-2^+^ cells expressing CD11b^+^Ly6G^−^. (**l**) Percentage of CD45^+^mKate-2^+^ cells expressing CD11b^+^Ly6G^+^. (**m**) Percentage of CD45^+^mKate-2^−^ cells expressing CD11b^+^Ly6G^−^. (**n**) Percentage of CD45^+^mKate-2^−^ cells expressing CD11b^+^Ly6G^+^. Negative control is represented in blue and inflammation-induced footpads, in red. Data are represented in bar graphs as the mean ± SD. Statistical significance was determined using *t*-test with Welch’s correction. *P < 0.05; **P < 0.01; ns = non-significant and *n* = 3 per group, done over one experiment.

The percentage of CD45^-^mKate-2^+^ cells co-expressing these markers was similar in footpads injected with either carrageenan or PBS (>70% in both groups) (Fig. 3a; representative gating in Supplementary Fig. S2a). However, the expression of Podoplanin (PDPN) on mKate-2^+^ cells increased following the induction of inflammation (Fig. 3b). Interestingly, in the mKate-2 negative stromal cell population (represented in Supplementary Fig. S2b), the percentage of cells expressing both markers was low in footpads injected with PBS and even lower in carrageenan-injected footpads (Fig. 3c); demonstrating that this phenotype is associated with *Col3a1* expression.

In the DTH model, the percentage of CD45^−^mKate-2^+^CD140a^+^PDPN^+^ cells were similar in both HAO and PBS-injected footpads (Fig. 3d; representative gating in Supplementary Fig. S2c). As observed in the carrageenan experiment, the expression of PDPN (MFI) was significantly higher in inflamed- (HAO) compared to PBS-injected footpads (Fig. 3e). The percentage of CD45^−^mKate-2^−^ cells expressing these markers was once again lower than that observed in mKate-2^+^ cells, confirming that cells co-expressing PDPN and CD140a are associated with *Col3a1* expression. There was no statistical difference between the studied groups (Fig. 3f, representative gating in Supplementary Fig. S2d).

### There is a bigger contribution of myeloid cells to *Col3a1* expression following inflammation

In addition to fibroblasts (Fig.s 2d and 2h) CD45^+^ cells expressed mKate-2 in both models, with increased percentages in inflamed footpads. In light of this interesting finding, we analysed the phenotype of these CD45^+^mKate-2^+^ cells, using the markers CD11b and Ly6G. Two populations of CD45^+^mKate-2^+^-expressing cells were identified: CD11b^+^Ly6G^−^ (monocytes/macrophages) and CD11b^+^Ly6G^+^ (neutrophils).

In the carrageenan model, there were differences in the proportion of cells depending on the treatment (representative flow gating in Supplementary Fig. S3a). Inflamed footpads had a higher frequency of monocytes/macrophages (Fig. 3g) and lower of neutrophils (Fig. 3h) expressing mKate-2^+^, compared to PBS-injected footpads. By comparison, in the mKate-2 negative population, there was no statistical difference between carrageenan- and PBS-injected footpads (Fig. 3i and j). Observing the flow cytometry plots for CD45^+^mkate-2^−^ cells, there was also a population of CD11b^−^Ly6G^−^ cells (mainly lymphoid cells) that are not present in the mKate-2^+^ population (represented in Supplementary Fig. S3b).

In the DTH experiment, almost 80% of the immune cells expressing *Col3a1* expression were CD11b^+^Ly6G^−^ in both the HAO and PBS-injected footpads (Fig. 3k; ns. Representative gating in Supplementary Fig. S3c), while the percentages of neutrophils were low (Fig. 3l; ns). When analysing CD45^+^mKate-2^−^ cells, both populations were present in similar frequencies (Fig. 3m and n, represented in Supplementary Fig. S3d); it was also possible to detect CD11b^−^Ly6G^−^ cells. In this model, no statistically significant differences in the mKate-2^+^ or mKate-2^−^ immune cells between PBS- and HAO-injected footpads were observed.

## Discussion

Although it is well documented that the upregulation of COL-3 gene expression is associated with early stages of fibrosis and joint-related diseases, until now, it was not possible to specifically track, identify and sort the cells expressing *Col3a1*. We, therefore, developed a reporter mouse in collaboration with GenOway that allows the examination *Col3a1*-expressing cells and how they change during inflammation and repair. We used two models of footpad inflammation to validate this reporter mouse. They were chosen as acute forms of inflammation with predominantly innate versus adaptive immune profiles and as precursors for disease-specific models.

It has been demonstrated previously that COL-3 is produced at the site of injury during tissue repair [15]. Indeed, for both models, the fluorescence increased with time, being higher 8–15 days after the initiation of inflammation. Thus, the *Col3a1*mKate-2 reporter mouse enables the specific detection and quantification of *Col3a1* expression in longitudinal studies, with a non-invasive whole-body imaging method. This is also advantageous in reducing in the number of mice per experiment, as the same mouse can be imaged at all timepoints. One limitation may be a detection of low and diffuse concentrations of mKate-2, but future work using models of chronic diseases should determine the utility to examine differences in the dynamics of *Col3a1* expression between normal repair and early stages of fibrosis.

This reporter mouse also allows the characterization of cells expressing *Col3a1*. Fibroblasts form a heterogeneous population of cells that could not be defined by one marker in specific.

With our reporter mice, we showed that most of the *Col3a1*-expressing stromal cells were CD140a and podoplanin-positive and that expression of PDPN increased after the induction of inflammation by carrageenan. The difference in podoplanin upregulation in both models could be an outcome of carrageenan driving a more severe inflammation. Fibroblasts expressing high levels of PDPN have already been associated with inflammatory processes and fibrosis [16]. Isolating these *Col3a1*^+^ cells in the future will allow us to investigate their transcriptional profile, to understand whether there is a definitive set of markers that identify them and whether there are differences in *Col3a1*-expressing cell phenotypes depending on the pathology. A similar process was described for a collagen type I reporter and demonstrated that, in pulmonary fibrosis, *Col1a*-expressing cells could be identified by the expression of *Cthrc1* (collagen triple helix repeat containing 1) and have a distinct location in the lung structure, exhibiting a more migratory behaviour, compared to other collagen-expressing cells [17]. It will be interesting to compare *Col3a1*- to *Col1a*-expressing cells, as their ratio seems to be a key factor during the onset of fibrosis.

Intriguingly, in our study, *Col3a1* was expressed not only by fibroblasts, but also by a population of CD45^*+*^ immune cells, and this population increased during inflammation. For both models, the main immune cells expressing *Col3a1* were CD11b^+^Ly6G^−^ (monocytes/macrophages). It has been reported previously that human monocytes and macrophages expressed fibronectin and all the main types of collagen mRNAs [18]. In this report, expression of *Col3a1* was low in comparison to other collagens. However, the cells examined were monocytes derived from the peripheral blood of healthy volunteers and macrophages differentiated *in vitro* from either these monocytes or a cell line [18]. Corroborating this hypothesis, COL-1-producing monocytes were found in the peripheral blood of systemic sclerosis patients [19]. Although macrophages are known for secreting proteases that degrade ECM, they may have a dual function, by also producing some types of collagens and other ECM components. Interestingly, collagen-producing macrophages seem to derive from an environment rich in regulatory cytokines, like IL-10 and TGF-β [18, 20]. Expanding our flow cytometry panel with more immune cell markers will provide more in-depth information about the phenotype and activation status of these CD11b^+^ immune cells expressing *Col3a1.* As we observed with the carrageenan and DTH models, it will be possible to detect differences within the populations depending on the nature of the inflammation.

In conclusion, homozygous *Col3a1*mKate-2 mice provided important information about *Col3a1*-expressing cell location, kinetics, and phenotype in two models of inflammation. Future work with this reporter mouse offers a spectrum of opportunities, where *Col3a1*-expressing cells could be isolated from different disease models for transcriptional profiling and for the development of *in vitro* models based on cell lines reporting the expression of *Col3a1*. Overall, we have demonstrated that our reporter mouse is a promising tool to investigate the onset of fibrotic processes, which has the potential to be used in a variety of pathologies.

## Supplementary Material

kyac008_suppl_Supplementary_Figure_S1

kyac008_suppl_Supplementary_Figure_S2

kyac008_suppl_Supplementary_Figure_S3

## Data Availability

The data underlying this article will be shared on reasonable request to the corresponding author.

## Abbreviations

CD: Cluster of differentiation
CFA: Complete Freund adjuvant
COL-3: Collagen type III
Cthrc1: Collagen triple helix repeat containing 1
DTH: delayed type hypersensitivity
ECM: extracellular matrix
FBS: foetal bovine serum
FGF: fibroblast growth factor
HAO: heat-aggregated OVA
IL: Interleukin
IRES: internal ribosome entry site
IVIS: *in vivo* imaging system
Ly6G: Lymphocyte antigen 6 complex locus G6D
MFI: median of fluorescence intensity
PBS: phosphate-buffered saline
PDGF: platelet-derived growth factor
PDPN: Podoplanin
RA: rheumatoid arthritis
ROI: region of interest
SD: standard deviation
SPF: specific pathogen free
SSc-ILD: sclerosis-associated interstitial lung disease
TGF-β: transforming growth factor-beta
WT: wildtype
