# Supplementary figures and images for: Revealing stromal and lymphoid sources of Col3a1-expression during inflammation using a novel reporter mouse
Source: Discov Immunol. 2022 Nov 21;1(1):kyac008. doi: 10.1093/discim/kyac008 (PMC10917174; doi:10.1093/discim/kyac008)

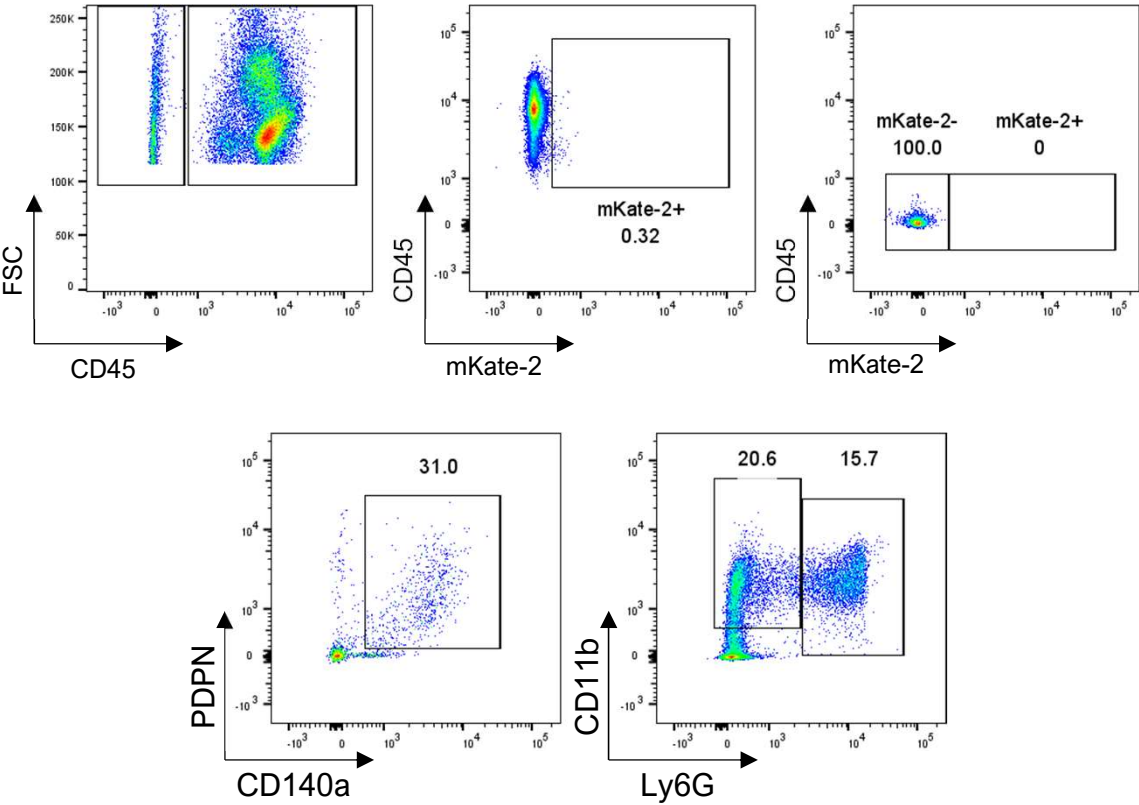

Supplement: kyac008_suppl_Supplementary_Figure_S1 [file kyac008_suppl_Supplementary_Figure_S1.pdf]

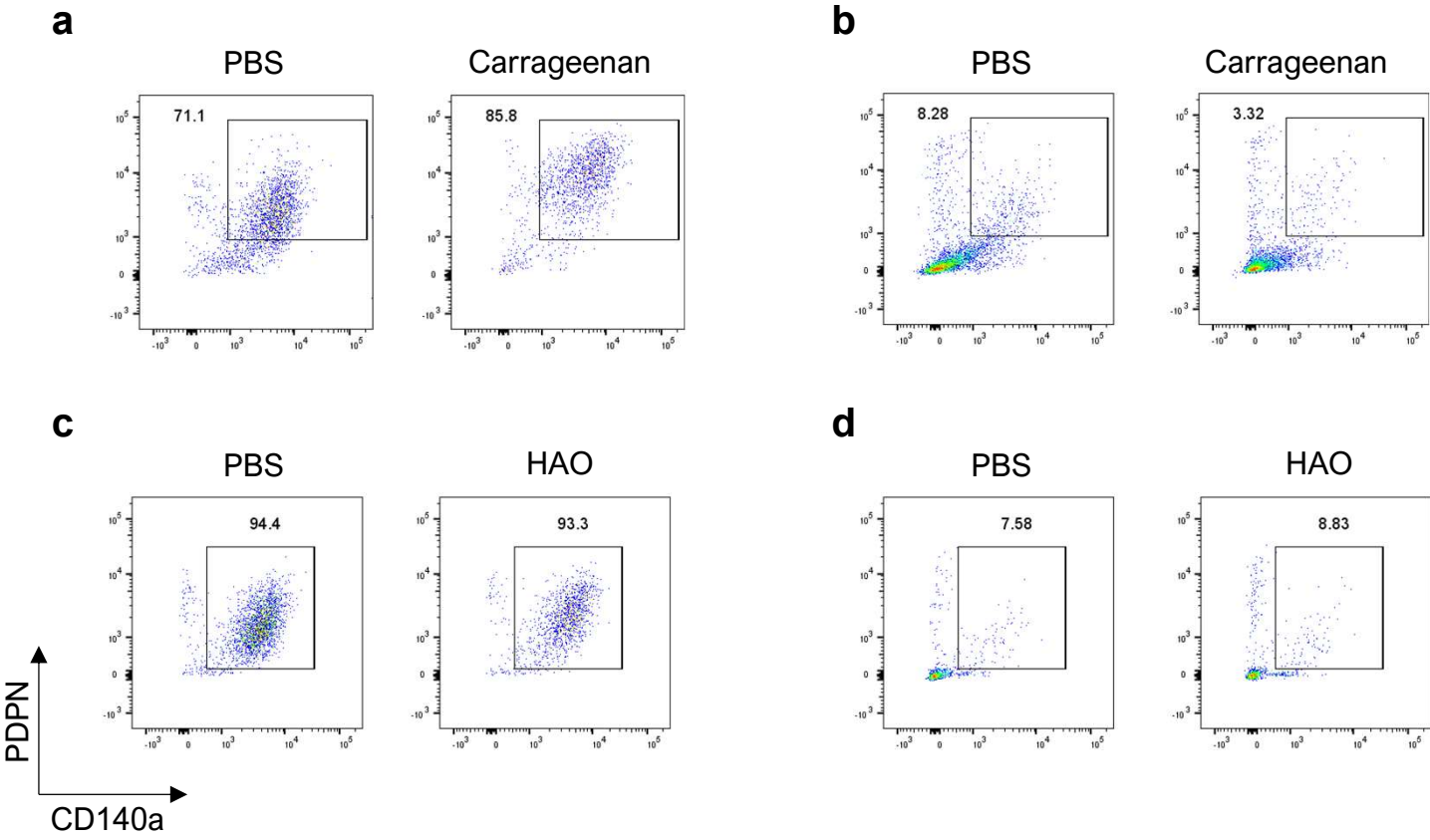

Supplement: kyac008_suppl_Supplementary_Figure_S2 [file kyac008_suppl_Supplementary_Figure_S2.pdf]

**a**

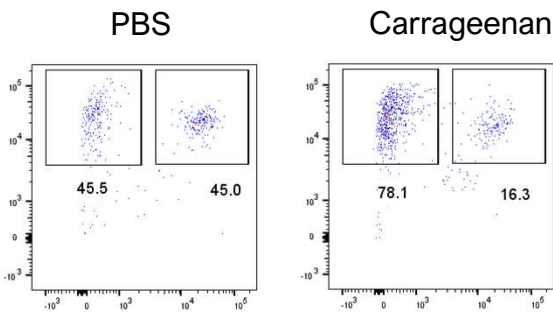

**b**

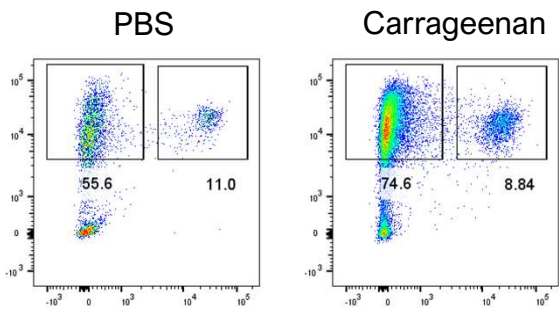

**c**

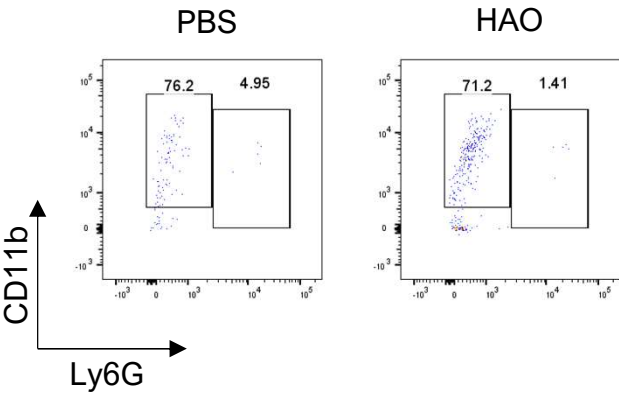

**d**

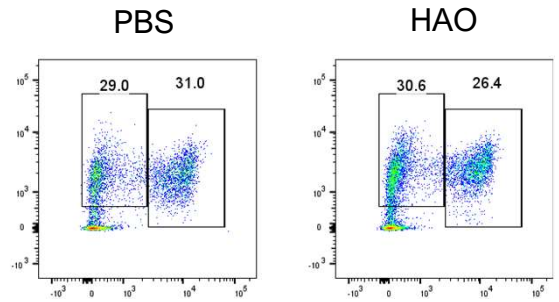

Supplement: kyac008_suppl_Supplementary_Figure_S3 [file kyac008_suppl_Supplementary_Figure_S3.pdf]
